# Effects of Weight Loss on Advanced Glycation End Products in Subjects with and without Diabetes: A Preliminary Report

**DOI:** 10.3390/ijerph14121553

**Published:** 2017-12-11

**Authors:** Permal Deo, Jennifer B. Keogh, Naomi J. Price, Peter M. Clifton

**Affiliations:** 1 Sansom Institute for Health Research, School of Pharmacy and Medical Sciences, University of South Australia, Adelaide, SA 5000, Australia; jennifer.keogh@unisa.edu.au; 2 School of Pharmacy and Medical Sciences, University of South Australia, Adelaide, SA 5000, Australia; naomi.joanne.price@hotmail.com

**Keywords:** advanced glycation end-products, *N*^ε^-carboxymethyllysine, weight loss

## Abstract

Advanced glycation end-products (AGEs) are formed endogenously as a normal ageing process and during food processing. High levels of AGEs have been implicated in the development of both macrovascular disease and microvascular disease. The purpose of this secondary analysis was to determine whether a major AGE species, *N*^ε^-carboxymethyllysine (CML), was reduced after weight loss. CML values decreased by 17% after weight loss. Participants with diabetes and pre-diabetes had a lower CML values at baseline and a smaller change in CML than overweight participants without diabetes. We conclude that, in addition to the known health benefits, weight loss may reduce AGEs. Randomized studies of the effect of weight loss on AGE in people with and without type 2 diabetes are needed to confirm these results.

## 1. Introduction

Advanced glycation end-products (AGEs) are the nonenzymatic posttranslational modification of carbonyl groups of reducing sugars and free amino groups of proteins [1]. AGEs are formed endogenously as a normal ageing process or during food processing and ingested via diet contributing to circulating and tissue AGEs in the body [2,3]. Consumption of high AGE food can increase the total daily AGE intake by 25% compared to the average adult intake [4,5]. It has been demonstrated that approximately 10–30% of dietary AGEs consumed are intestinally absorbed, with only one-third of ingested AGEs excreted in urine and faeces [6]. *N*^ε^-carboxymethyllysine (CML), a major AGE species, has frequently been used as a marker both in foods and *in vivo* [7,8,9]. The absorption of CML from foods, its metabolism, distribution, and elimination are partly elucidated [10] and a subject of current research interest [11,12]. Excessive AGE accumulation in the body may cause significant cellular dysfunction and are implicated in the development of both macrovascular disease and microvascular diseases in people with diabetes [13]. The mechanisms involved are thought to be increased vascular and myocardial stiffening, inflammation, and oxidative stress [14].

The effects of decreasing the dietary intake of AGEs on risk markers for cardiovascular disease has been examined in a recent meta-analysis of randomized controlled trials that included 560 participants [15]. Low AGE diets were found to decrease insulin resistance in the whole group, decrease total and low density lipoprotein (LDL)-cholesterol in those without diabetes, and decrease fasting insulin and C-reactive protein (CRP) in people with type 2 diabetes (n = 112). Reductions in tumour necrosis factor α (TNF-α), vascular cell adhesion molecule-1 (VCAM-1), 8-isoprostane, leptin, circulating AGEs, and soluble receptor for AGEs were reported. An increase in adiponectin, white cell sirtuin-1 mRNA, and the estimated glomerular filtration rate were also reported. Kellow (2013) also reported benefits of AGE reduction in a review of 12 trials with 289 participants [16]. Meta-analysis of two 16-week trials showed a reduction in 8-isoprostanes and TNF-α. There were beneficial effects on VCAM-1 in people with chronic renal failure and on the homeostatic model assessment of insulin resistance and LDL-cholesterol in people without diabetes. Clarke (2016) identified 12 dietary AGE intervention studies with 293 participants and reported that a high AGE intake increased TNF-α in all populations and increased 8-isoprostanes in healthy subjects and VCAM-1 in people with diabetes [3]. However, the current studies lack enough high-quality randomized trials to make a recommendation that dietary AGE restriction would alleviate chronic disease such as inflammation and oxidative stress.

Foods high in protein and fat have the highest amounts of AGEs, and foods that have been cooked at high temperature, e.g., fried, grilled, or roasted, will have higher AGEs than foods boiled, poached, or stewed [14]. Intervention studies of low AGE diets provide limited information on how the dietary change was achieved, so it is unclear if cooking methods were changed or if particular foods were excluded or both [5,17].

Energy restriction for weight loss is likely to result in a decreased intake of AGEs, as high-energy foods cooked at high temperatures, e.g. roasted nuts and fried food, as well as fat intake will be reduced. Gugliucci (2009) found that serum AGEs were reduced by 7% after weight loss in 37 subjects [18]. However, AGEs were measured by fluorescence intensity rather than by measurement of specific AGE species such as *N*^ε^-carboxymethyllysine (CML), a stable, relatively inert and non-fluorescent AGE [19].

The purpose of this secondary analysis was to determine whether a major AGE species, *N*^ε^-carboxymethyllysine (CML) was reduced after weight loss in subjects with and without diabetes who had participated in previously published weight loss studies [20,21].

## 2. Materials and Methods

### 2.1. Study Participants

In the first weight loss study, 120 men were randomized to a low-fat, energy-restricted diet (7 MJ/day) either high in protein (HP) or carbohydrate (HC) for 52 weeks, and 68 completed the study (33 HP, 35 HC). Both groups lost weight (HP −12.3 ± 8.0 kg; HC −10.9 ± 8.6 kg) indicating adherence to the prescribed energy reduction [20]. In the second study, 65 participants with type 2 diabetes or impaired glucose tolerance were randomized to two energy-restricted diets (6–7 MJ/day) that differed in cholesterol content (590 mg or 213 mg) for 12 weeks [21]. Overall weight loss was 6.0 ± 0.4 kg, indicating adherence to the prescribed energy reduction. In both studies, serum samples were isolated and frozen at −80 ± 1 °C until used for AGE analysis. Both studies had ethics approval from the human ethics committees of the Commonwealth Scientific and Industrial Research Organization. All participants provided written informed consent before commencement.

### 2.2. Biochemical Analysis

The data for total cholesterol, LDL-cholesterol, high-density lipoprotein (HDL)-cholesterol, triglycerides, glucose, and glycosylated haemoglobin A1c (HbA1c) were obtained from previous studies [20,21].

### 2.3. CML Analysis

#### 2.3.1. Sample Preparation

Serum (100 µL) was prepared by reduction with sodium borohydride and protein isolation with trichloroacetic acid (20%) and then hydrolyzed with hydrochloric acid (6 M) at 110 ± 1 °C for 24 h as previously described [2]. Each protein hydrolysate was subjected to solid phase extraction (SPE) using preconditioned Supelco C18 cartridge (Sigma, St. Louis, MO, USA). The analyte of interest was eluted with 1% trifluoroacetic acid in methanol/water (20:80, 3 mL), dried under vacuum, and reconstituted in acetonitrile (50%, 200 µL) prior to analysis.

#### 2.3.2. CML Quantification Using RP-HPLC

Prior to HPLC injections, samples were derivatized with o-phthaldialdehyde/*N*-acetyl-l-cysteine (OPA/NAC) as previously described [22]. Derivatized samples were injected (15 µL) onto an RP C18 HPLC column (Aeris®, 2.1 × 150 mm, particle size 3.6 µm, pore size 200 Å; Phenomenex, NSW, Australia). The column was heated to 37 ± 1 °C, and the sample was monitored with a fluorescence detector at 340 nm Ex/450 nm Em. Acetonitrile (100%) was used as the mobile phase at a flow rate of 0.7 mL/min over 10 min. Lysine (Lys) standards were also derivatized with OPA/NAC as above before injection. The concentration of Lys and CML were determined from a 5-point calibration standard curve of lysine (Sigma, St. Louis, MO, USA) and CML (Polypeptide Laboratories, France), respectively. The standards were also subjected to SPE and measured in triplicates, while samples were measured in duplicates. Results are presented as mmol CML/mol Lys.

### 2.4. Statistics

Data was analyzed using a Repeated Measures ANOVA in SPSS v22 (IBM, Armonk, NY, USA). *p* < 0.05 was accepted as significant.

## 3. Results

### 3.1. The Effect of Weight Loss on Biochemical Characteristics and CML Levels

**Figure 1:**
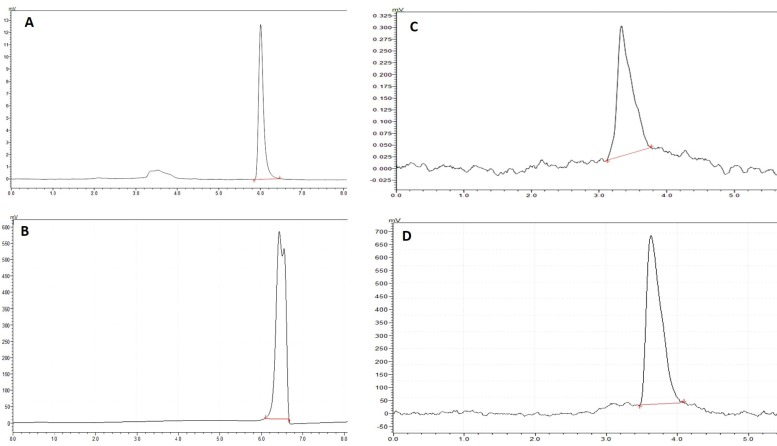
HPLC chromatographs of OPA/NAC derivatized standards and samples. (**A**) Lysine standards (0.25 mmol/L); (**B**) Lysine detection in plasma protein (1:500 dilution); (**C**) *N*^ε^-carboxymethyllysine (CML) standards (0.25 mmol/L); (**D**) CML detection in plasma protein.

Samples were available from 49 (31 male, 18 female) participants who were 57 ± 9 years, had a BMI of 32.7 ± 6.8 kg/m^2^ and lost 7.9 ± 4.1 kg of body weight. Total cholesterol, triglycerides, and glucose were significantly decreased in these subjects, whereas no significant changes were seen in terms of LDL-cholesterol and HDL-cholesterol (Table 1). HbA1c, an indicator of plasma protein glycation, was significantly reduced (6.8 ± 0.7 to 6.2 ± 0.5, *p* < 0.001). CML values, a ratio between CML and lysine after derivatization, in plasma are reported as mmol CML/mol Lys as previously reported [23,24]. Figure 1 represents an HPLC chromatogram of lysine and CML in standards and a sample. CML values decreased by 17% after weight loss (Table 1). Participants with diabetes and pre-diabetes had a lower CML at baseline (0.062 ± 0.009 vs. 0.081 ± 0.022 mmol CML/mol Lys *p* < 0.01) and a smaller change in CML than overweight participants without diabetes (0.005 ± 0.015 vs. 0.017 ± 0.020 mmol CML/mol Lys, *p* < 0.01). These differences were independent of gender, age, weight at baseline, and weight loss.

### 3.2. Correlation Analysis

The correlation analysis indicated a weak but significant relationship between weight change and change in HbA1c (−0.33, *p* < 0.05). However, no correlations between HbA1c and CML either before (n = 35) or after weight loss (n = 30) or between CML change and change in HbA1c or weight change and CML were observed.

## 4. Discussion

In this study, both HbA1c and CML significantly decreased after weight loss, suggesting the possibility that energy restriction reduces AGE intake and thus lowers CML levels. However, AGE levels in the diet were not measured, as the study was focused on macronutrient composition for energy restriction. In this study, a weak but significant relationship was observed between weight loss and HbA1c as in the previous report [25]. Plasma AGE concentration appears to be influenced by dietary AGE intake, but previous studies have shown conflicting data on the relationship between dietary AGE consumption and circulating CML levels [5,26,27,28,29].

As in the present study, Gugliucci and coworkers [18] found that AGEs were reduced after weight loss but used plasma fluorescence to measure them rather than a more robust CML measure as used in the present analysis. A low energy low AGE Mediterranean diet has been shown to decrease CML levels by >30% in a single arm study after 3 kg of weight loss. Dietary quality as shown by an increase in the Mediterranean Diet Score improved, but AGE intake was not calculated before or after the diet [30]. In contrast, Sánchez and coworkers found that AGEs measured using skin autofluorescence did not decrease following weight loss when measured five years after bariatric surgery [31]. However, the authors suggest that this result was not unexpected, as protein turnover is a major determinant of AGE accumulation in collagen and may be take up to 15 years [32], so the timeframe of the study was not sufficient to see a decrease. In women with and without polycystic ovary syndrome, both orlistat and a low-calorie diet decreased weight and AGE with an improvement in insulin resistance after 6 months [33]. In these previous studies, AGEs/CML levels were reported based on either fluorescence or immunoassays that yield only semi-quantitative results with uncertainty on the specificity of the antibody used. In our study, CML, a specific AGE, was detected and quantified in plasma proteins using analytical techniques. It is noteworthy that the participants with diabetes and pre-diabetes had a lower CML at baseline and a smaller but significant change in CML after 12-week weight loss. Two homogenous trials involving long-term (6–16 weeks) interventions showed that low-AGE diets reduced circulating CML concentrations in adults with type 2 diabetes [5,28]. In addition, our studies showed significant reduction in CML values in overweight but otherwise healthy adults when subjected to 52 weeks of energy restricted diet. Long-term (16 weeks) dietary AGE restriction significantly reduced circulating CML concentration in healthy adults [5,29]. Once again, it is worth mentioning that CML concentrations in most of these previous studies were based on immunological assays.

In this study, protein-bound plasma CML values were significantly reduced after weight loss. As our study did not measure dietary CML levels, we are not certain that the reduction in plasma CML values was achieved through a dietary reduction in AGE. Previous studies have shown that protein-bound plasma CML may not be influenced by dietary AGEs [26,27,34], although the data are conflicting (e.g., Uribarri et al., [35,36]), thus suggesting that another mechanism may be responsible for the reduction in CML after weight loss. AGEs are generated *in vivo* as a normal process of metabolism, but their formation is accelerated under conditions of hyperglycemia, hyperlipidemia, and increased oxidative stress. Thus, CML could be generated through different pathways, including fructosamine oxidation and reaction with lipid peroxidation-derived reaction products [9,37]. Other investigations on energy restriction for weight loss have also shown significant reduction in lipid peroxidation biomarkers, including malondialdehyde and isoprostane, suggesting a decrease in oxidative stress status [25,38,39]. Taken together, it can be speculated that weight loss as seen in our studies may have reduced the probability of lipid-derived CML formation and reduced oxidative stress and eventually led to less CML.

Reducing dietary intake of AGE may improve risk markers for cardiovascular disease as shown in a recent meta-analysis of randomized controlled trials in which low AGE diets decreased insulin resistance overall, decreased total and LDL-cholesterol in those without diabetes, and decreased fasting insulin and C-reactive protein in people with type 2 diabetes [15]. Most of the studies included in this meta-analysis are confounded on differences in the diet, disease state of the patients and AGE species. In addition, dietary quality may be improved during weight loss as participants may increase intake of fruits and vegetables and reduce fat intake [40]. However, improving dietary quality alone did not reduce CML levels or plasma fluorescent AGEs despite an improvement in insulin sensitivity [41] and a marked reduction in plasminogen activator inhibitor-1, which has been linked to serum AGE levels [42].

The finding that participants with diabetes and pre-diabetes had a lower CML at baseline and a smaller change in CML after weight loss was unexpected, and it is unclear as to why this may have happened.

## 5. Conclusions

We conclude that weight loss appears to decrease AGE as measured by CML in overweight men and to a lesser extent men and women with impaired glucose and type 2 diabetes. There is a clear need for larger randomized studies of the effect of weight loss on different AGE species in people with and without type 2 diabetes.

## Figures and Tables

**Table 1:** The effect of weight loss on biochemical characteristics and CML values.

Parameters	n	Before Weight Loss	After Weight Loss	*p*-Value
TC (mmol/L)	39	4.9 ± 0.9	4.5 ± 0.9	<0.01
TG (mmol/L)	39	1.8 ± 0.8	1.4 ± 0.6	<0.001
HDL-cholesterol (mmol/L)	39	1.2 ± 0.3	1.3 ± 0.3	>0.05
LDL-cholesterol (mmol/L)	39	2.9 ± 0.8	2.7 ± 0.8	>0.05
Glucose (mmol/L)	41	6.8 ± 1.7	6.3 ± 1.1	<0.05
HbA1c (%)	29	6.8 ± 0.7	6.2 ± 0.5	<0.001
CML (mmol CML/mol Lys)	49	0.070 ± 0.017	0.060 ± 0.009	<0.001

Data are presented as mean ± SD. Total cholesterol (TC), triglycerides (TG), high-density lipoprotein cholesterol (HDL-cholesterol), low-density lipoprotein cholesterol (LDL-cholesterol), glycosylated haemoglobin A1c (HbA1c), *N*^ε^-carboxymethyllysine (CML).
